# Achieving Ultra‐High Heat Flux Transfer in Graphene Films via Tunable Gas Escape Channels

**DOI:** 10.1002/advs.202410913

**Published:** 2024-11-11

**Authors:** Haolong Zheng, Peng He, Shujing Yang, Yonghua Lu, Na Guo, Yanhong Li, Gang Wang, Guqiao Ding

**Affiliations:** ^1^ State Key Laboratory of Materials for Integrated Circuits Shanghai Institute of Microsystem and Information Technology Chinese Academy of Sciences Shanghai 200050 P. R. China; ^2^ College of Materials Science and Opto‐Electronic Technology University of Chinese Academy of Sciences Beijing 100049 P. R. China; ^3^ Zhongke Yueda Shanghai Material Technology Co. Ltd Shanghai 201800 P. R. China; ^4^ School of Physical Science and Technology Ningbo University Ningbo 315211 P. R. China

**Keywords:** gas escape channels, graphene films, heat dissipation, highly thermally conductive, structural regulation

## Abstract

Graphene films have been applied in the thermal management of electronic devices due to their high thermal conductivity. However, the ever‐increasing power and local heat flux density of electronic chips require graphene films with excellent heat flux carrying capacity. Enhancing the heat flux carrying capacity is highly challenging, and the key is to maintain high thermal conductivity while increasing film thickness. Gases released during film assembly and the resulting catastrophic structural destruction should be responsible for the trade‐off between film thickness and thermal conductivity. Herein, the evolution of the pore structure is investigated during the assembly of graphene films and propose the construction of gas escape channels for the preparation of thick graphene films. The process involves using humidification treatment and freeze‐drying GO films to pre‐construct the ordered flat pore structure. The microstructure optimization of graphene films with more order, fewer wrinkles and defects, and larger grain size is achieved. After optimization, graphene films with ultra‐high thermal conductivity (1781 W m^−1^ K^−1^) and a thickness over 100 µm are realized. These films exhibit exceptional heat dissipation and cooling capabilities in high heat flux density (≈2000 W cm^−2^). This finding holds significant potential for guiding the thermal management of high‐power devices.

## Graphical abstract

The pre‐built flat and well‐organized gas escape channel helps reduce structural damage caused by exhaust gases during the heat treatment of graphene films. This enables the preparation of graphene films with high thermal conductivity and substantial thickness, making them suitable for dissipating heat under high heat flux.

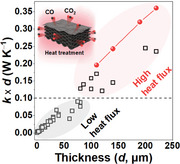

## 1 Introduction

When developing highly integrated and high‐power electronic devices, managing the ever‐increasing localized heat generation is crucial. Failure to effectively manage this heat can critically undermine the stable operation of the devices. Therefore, extensive attention has been paid to developing heat‐dissipation materials with high thermal conductivity.^[^ ^1^, ^2^ ^]^ These materials are designed to quickly dissipate localized heat, thereby improving the cooling efficiency of the devices. The heat flux carrying capacity, which reflects thermal conductivity and material thickness, is increasingly important in practical applications. Graphene is a promising candidate for high heat flux carrying capacity, given its exceptional thermal conductivity of ≈5000 W m^−1^ K^−1^ at room temperature.^[^ ^3^, ^4^ ^]^ The thermal conductivity of graphene films with thicknesses ranging from tens of nanometers to a few micrometers has also reached impressive values in the range of 2000–4000 W m^−1^ K^−1^.^[^ ^5^, ^6^, ^7^, ^8^ ^]^ Compared with traditional heat spreaders (e.g., copper foils, natural graphite films, and artificial graphite films), graphene films assembled from graphene‐based materials offer superior performance, including high flexibility, thermal conductivity, and lightweight.^[^ ^9^, ^10^ ^]^ However, improving heat flux carrying capacity is hindered by the sharp decrease in thermal conductivity with increasing thickness due to the extreme phonon dispersion anisotropy and structural defects in graphene films.^[^ ^8^, ^11^ ^]^ Reported work indicates that the thermal conductivity of graphene films over a hundred microns thick is still suboptimal.

Achieving graphene films with high heat flux carrying capacity requires resolving the challenge posed by the trade‐off between high thickness and low thermal conductivity. One of the main factors contributing to this trade‐off is using graphene oxide (GO) to fabricate thermally conductive graphene films. GO is a chemical derivative of graphene with a high oxygen content of ≈30%, which offers good processability^[^ ^12^, ^13^ ^]^ but exhibits low thermal conductivity.^[^ ^14^, ^15^ ^]^ The process of preparing graphene films from GO involves film assembly, heat treatment, and mechanical compression. High‐temperature heat annealing effectively removes oxygen‐containing functional groups from GO and reinstates *sp* ^2^ domains in graphene films, a crucial step in enhancing thermal conductivity.^[^ ^16^ ^]^ However, the removal of these oxygen‐containing functional groups during the reduction of GO leads to the release of gases (e.g., H_2_O, CO, and CO_2_),^[^ ^17^ ^]^ resulting in the production of ≈0.2 L of gas per gram of GO and a mass loss of ≈40 wt %.^[^ ^18^ ^]^ The gas production rate surpasses the rate of gas elimination, causing gas accumulation and formation of gas pockets within the film.^[^ ^19^ ^]^ These gas pockets impede phonon transport, leading to decreased thermal conductivity.^[^ ^20^ ^]^ As the thickness of the film increases, the release of gas from the interior becomes constrained, potentially damaging the original well‐ordered, stacked structure of the GO film and even causing film rupture. The thermal conductivity of graphene film decreases sharply with increasing film thickness.^[^ ^8^, ^21^ ^]^ Currently, thick graphene films are generally prepared by laminating multiple thin films,^[^ ^22^, ^23^, ^24^, ^25^, ^26^ ^]^ but the presence of gaps between the layers impedes the heat transfer. To eliminate layer gaps, graphene films with a thickness of up to 200 µm and a thermal conductivity of 1224 W m^−1^ K^−1^ were successfully prepared by self‐fusing multiple GO films in water.^[^ ^27^ ^]^ However, the adverse effects of releasing heat‐treated gases remain unmitigated. To minimize gas produced during heat treatment, Zhu et al. developed a method involving ascorbic acid to pre‐reduce the oxygen‐containing functional groups in GO. This resulted in the production of graphene films with a thickness of 80 µm and a remarkable thermal conductivity of up to 1600 W m^−1^ K^−1^.^[^ ^28^ ^]^ Ascorbic acid was found to gently reduce the oxygen‐containing functional groups of GO, thereby decreasing the gas generated during subsequent heat treatment. Furthermore, introducing the ascorbic acid derivatives was observed to connect the small graphene sheets, repair defects, cross‐link, and fuse the multilayered graphene films during hot‐pressing.^[^ ^25^ ^]^ The introduction of polyacrylonitrile into GO allowed for the release of free gas generated during the heat annealing, thus facilitating the transformation into an intact graphene lattice.^[^ ^29^ ^]^ Nevertheless, incorporating polymers into GO films to construct gas escape channels may introduce additional heteroatoms, which could adversely affect the processability of GO dispersion. Achieving graphene films with both high thermal conductivity and substantial thickness has proven to be quite challenging. Therefore, mitigating the adverse effects of gas release from heat treatment and improving the thermal conductivity of thick graphene films have attracted increasing attention, particularly for heat dissipation in high‐power electronic devices.

Herein, we propose a new strategy for constructing gas escape channels within GO films to solve the critical problem of heat‐transfer properties degradation caused by the obstructed release of gases. This method facilitates the elimination of gases generated during heat treatment, producing thick graphene films with low wrinkle density and high orientation. A porous structure as gas escape channels is constructed between GO films through water absorption and freeze‐drying treatment, increasing the porosity from 11.01 to 20.79%, and creating a uniform and ordered distribution structure. Then, the GO film with a porous structure undergoes heat treatment, allowing the gases produced from the removal of oxygen‐containing functional groups to escape through the pre‐constructed pore channels, thus mitigating the adverse effects of gas impingement on the graphene film. This process yields graphene films with high orientation (*f* = 0.959), low wrinkle density (*ρ*_w_ = 99 mm mm^−2^), low defects (*I*_D_/*I*_G_ = 0.012), and large grain size (*L*_c_ = 44.5 nm). Compared with the graphene films prepared by the conventional route, the thermal conductivity of the obtained thick films (≈110 µm thick) improves by 16.2% to ≈1781 W m^−1^ K^−1^, which possesses a high heat flux carrying capacity. Under a heat flux density of ≈2000 W cm^−2^, the temperature of a chip attached to a graphene film can be effectively reduced by up to 16 °C, and a uniform temperature distribution across the film surface.

## 2 Results and Discussion

### 2.1 Preparation and Thermal Properties of Graphene Films

**Figure 1:**
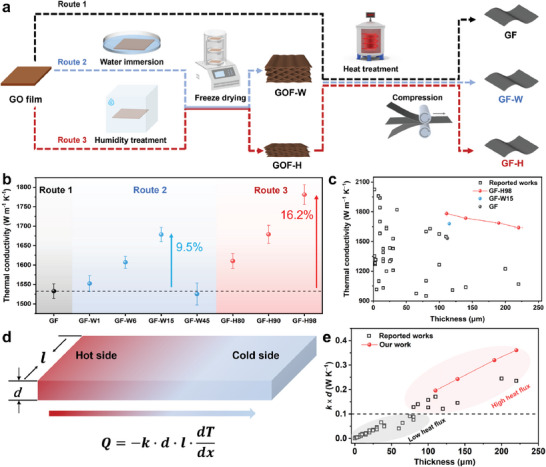
Preparation of high thermal conductive graphene films by constructing gas escape channels in GO films. a) Scheme for preparing the graphene films via different routes. b) Comparison of thermal conductivity of graphene films prepared by various routes. c) Comparison in thermal conductivity and film thickness of GF, GF‐W15, GF‐H98, and reported works. d) Schematic model of heat transfer and Fourier's law. e) Comparison in *k* × *d* values of GF‐H98 and reported works.

GO served as the assembly unit, and the GO films with a layered structure were prepared using a doctor‐blade coating. Heat annealing is essential to eliminate oxygen functional groups in GO films and form the desired lattice structure for thermal transfer.^[^ ^30^ ^]^ The conventional process for preparing graphene films (GF) involves direct heat treatment and compression (Route 1). Water immersion and lyophilization (Route 2), humidification, and lyophilization (Route 3) of GO film can pre‐construct porous structures to inhibit film expansion during heat treatment (**Figure** **1**a). The dry, untreated GO film is freeze‐dried to confirm that the film with no free water is unaffected at the freeze‐drying stage (Section S1, Figure S1, Supporting Information). The gases generated within the film during the heat treatment disrupt the alignment of the graphene sheets and are challenging to eliminate using conventional methods. Fine‐tuning the structure of GO films may lead to a reduction in structural defects during heat treatment. As shown in Figure 1b, the thermal conductivity (Section S2.2, Supporting Information) of the graphene films prepared by the conventional method (Route 1) is 1533 ± 18 W m^−1^ K^−1^. When a pre‐construction process is carried out to create a gas escape channel by immersing GO films in water for 15 min followed by freeze‐drying (Route 2), the resulting thermal conductivity of GF‐W15 increases by 9.5%–1678 ± 18 W m^−1^ K^−1^. Further increase in immersion time to 45 min leads to a decrease in the thermal conductivity of the samples, which is attributed to the disruption of the film structure caused by excessive water (Figure S2, Supporting Information). Surprisingly, the humidification treatment can regulate the water absorption of the GO film and form relatively ordered porous channels inside the film after freeze drying (Route 3), which facilitates the release of gases during heat treatment. Upon humidification treatment at 98% humidity and freeze‐drying, the resulting GF‐H98 demonstrates an improved (16.2%) thermal conductivity of 1781 ± 25 W m^−1^ K^−1^. Compared with previously reported work,^[^ ^31^, ^32^, ^33^, ^34^, ^35^, ^36^, ^37^, ^38^, ^39^, ^40^, ^41^, ^42^, ^43^ ^]^ the thermal conductivity of GF‐H98 with high thickness, offers a significant advantage, as depicted in Figure 1c (Table S1, Supporting Information). It's worth noting that film thickness also plays a crucial role in heat flux transportation for graphene film. According to Fourier's law,^[^ ^44^ ^]^ the total conductive heat (*Q*) can be calculated using the Equation (1):

(1) $$Q=k\cdot d\cdot l\cdot \frac{dT}{dx}$$

where Q is proportional to the thermal conductivity (*k*) and thickness (*d*) under a given transverse width size (*l*) and temperature gradient ($\frac{dT}{dx}$) (Figure 1d). The value of *k* × *d* is commonly used to evaluate the heat flux carrying capacity of thermally conductive graphene films,^[^ ^25^, ^35^ ^]^ which is a significant concern for ultrahigh heat flux‐related applications. Figure 1e summarizes the *k* × *d* values of recently reported graphene films prepared by various methods. The GF‐H98 exhibits a higher capacity for carrying heat flux during practical applications of heat dissipation. Therefore, the proposed strategy offers a notable advantage in preparing graphene films with high heat flux carrying capacity.

### 2.2 Construction of Porous Structure of Precursor GO Films

**Figure 2:**
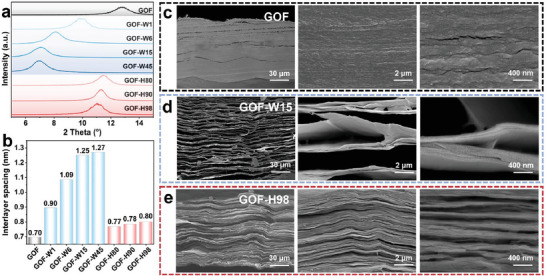
Evolution of GO film structure in different routes. a) XRD spectra of GO films with different routes. b) Histogram of layer spacing in GO films under different treatment conditions. Typical Cryo‐SEM images with different magnifications of the cross‐section in GO films: c) GOF d) GOF‐W15 e) GOF‐H98.

The GO films contain many hydrophilic oxygen‐containing functional groups, allowing them to absorb water molecules from the environment.^[^ ^45^ ^]^ As the quantity of absorbed water molecules increases, a network channel between the GO sheets forms, expanding the layer spacing of GO films.^[^ ^46^ ^]^ This expansion in layer spacing was accomplished through water immersion or humidification treatment, resulting in a rich pore structure within the films after freeze‐drying. Compared with the untreated GO films (GOF), it is evident that the interlayer spacing of the GO films after water immersion (GOF‐W) or humidification treatment (GOF‐H) increases obviously, as depicted in **Figure** **2**a. As shown in Figure 2b, the interlayer spacing of the GO films increased from 0.7 to 1.27 nm as the water immersion time increased from 1 to 45 min. In addition, the interlayer spacing of GOF‐80, GOF‐H90, and GOF‐H98 was measured at 0.77, 0.78, and 0.8 nm respectively, under humidification treatment conditions of 80, 90, and 98% (Table S2, Supporting Information). Upon immersion or humidification treatment, the water adsorbed within the GO films was rapidly removed through freeze‐drying. The cross‐sectional morphology of the GO films was examined using cryo‐scanning electron microscopy (Cryo‐SEM). The GOF obtained by directly drying without any treatment shows a tightly packed lamellar structure with a few pores between the layers (Figure 2c). The structural evolution of GO films after water immersion for 15 min and humidification treatment at 98% was studied, focusing on the excellent thermal conductivity of GF‐W15 and GF‐H98. Following water immersion and freeze‐drying, GOF‐W15 exhibited a loose porous structure (Figure 2d). In contrast, humidification treatment reduced water absorption and inhibited the formation of loose pores, resulting in GOF‐H98 with a relatively smaller and flatter pore structure after freeze‐drying (Figure 2e).

**Figure 3:**
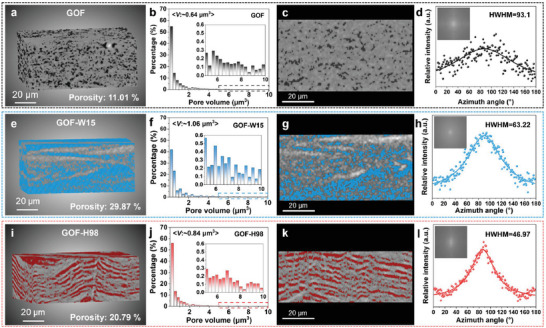
Characterization and analysis of porous structures in GO films. The 3D reconstruction images, the voids volume distributions, the cross‐section reconstruction images, and the angular analysis fitted with the Cauchy–Lorentz distribution (illustration is FFT frequency domain images) of GO films: a–d) GOF, e–h) GOF‐W15, and i–l) GOF‐H98.

The lamellar film formed by assembling 2D GO sheets naturally contains some pores with a porosity of ≈10%.^[^ ^47^ ^]^ To examine the pore structure within the films in detail, 3D X‐ray diffraction microscopy (XRM) was used to GO films under different treatment conditions. 3D reconstruction of the sample images revealed that the porosities of GOF were 11.01%. The pores were found to be dispersed randomly and independently throughout the film (**Figure** **3**a). The distribution of pore volume is depicted in Figure 3b, showing that the majority of the pores are within the range of less than 2 µm^3^, with an average pore volume of only 0.64 µm^3^. To assess the alignment of the interlayer pores within the reconstructed cross‐sectional image of the GOF, a fast Fourier transform (FFT) method was employed (Section S2.3, Supporting Information). This method involves converting the gray values of each image pixel into directional frequency components and analyzing the intensity of each pixel in relation to its spatial arrangement pattern.^[^ ^48^ ^]^ The FFT analysis was conducted on the cross‐sectional reconstructed image of the GOF (Figure 3c) using Image J. The resulting FFT frequency domain image, as depicted in Figure 3d, along with the corresponding angular analysis, revealed a half width at half maximum (HWHM) of 93.1, indicating that the pores are randomly and disorderly distributed in the horizontal direction of the film. Following water immersion treatment, the GO film was found to adsorb a significant amount of water, leading to the formation of partially interconnected large pores after freeze‐drying and achieving a porosity of up to 29.87% (Figure 3e). Therefore, the average pore volume of GOF‐W15 is increased to 1.06 µm^3^, accompanied by a relatively higher percentage of large pores (Figure 3f). Upon analyzing the cross‐sectional reconstructed image of the GOF‐W15, as shown in Figure 3g, it was found that the HWHM value reduced to 63.22, indicating a more favorable orientation of pore distribution compared to the original GOF (Figure 3h). The humidification treatment enables the GO film to maintain a relatively low water absorption, allowing water to accumulate horizontally along the film and forming flat pores after freeze‐drying. As depicted in Figure 3i, the porosity of GOF‐H98 obtained by humidification treatment at 98% is between GOF and GOF‐W15 at 20.79%. The percentage of large pores in GOF‐H98 decreased, with an average pore volume of 0.84 µm^3^ (Figure 3j). Notably, the cross‐sectional reconstructed image of GOF‐H98 (Figure 3k) exhibited the lowest HWHM value of 46.97 after FFT and angular analysis, suggesting an optimal orientation of pore distribution in the horizontal direction of the film (Figure 3l). These results demonstrate that immersion in water or humidification treatment followed by freeze‐drying effectively constructs a porous structure within the GO film, providing a pathway for the release of gases during the subsequent heat treatment.

### 2.3 Evolution of Surface Morphology and Orientation of Graphene Films

**Figure 4:**
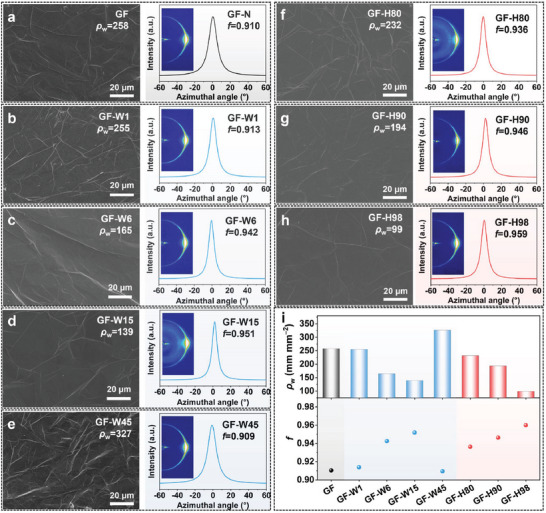
Surface morphology and orientation of graphene films. Typical SEM images of surface in graphene films, the WAXS pattern, and the corresponding azimuthal scan profile of graphene films: a) GF, b) GF‐W1, c) GF‐W6, d) GF‐W15, e) GF‐W45, f) GF‐H80, g) GF‐H90, and h) GF‐H98. i) Statistics of the density of surface wrinkles (*ρ*_w_) and orientation factor (*f*) of graphene films.

The GO films obtained under various conditions underwent segmented heat treatment and subsequent mechanical compression. The untreated GO films, those immersed in water for different durations followed by freeze‐drying, and those subjected to humidification treatment at varying humidity levels followed by freeze‐drying were all heat annealed and then mechanically compressed to produce dense graphene films. The surface morphology and orientation of the graphene films were characterized using SEM and wide‐angle X‐ray scattering (WAXS) (Section S2.4, Supporting Information). Wrinkling is typical in 2D films, with high wrinkling density (*ρ*_w_) typically observed in assembled graphene films comprising numerous sheets.^[^ ^49^ ^]^ The orientation factor (*f*) derived from the WAXS method is commonly used to measure sheet alignment.^[^ ^47^, ^50^, ^51^ ^]^ The formation of air pockets within the film, attributed to gases generated during heat treatment, is a primary cause of wrinkles and misalignment in graphene films following mechanical compression. The analysis in **Figure** **4**a reveals that the graphene film surface is characterized by numerous wrinkles, and the *ρ*_w_ of 258 mm mm^−2^ and the *f* of 0.91. Upon increasing the water immersion time from 1 to 15 min, the *ρ*_w_ for GF‐W1, GF‐W6, and GF‐W15 decreased from 255 to 139 mm mm^−2^, while the *f* of the films increased from 0.913 to 0.951 (Figure 4b–d). This indicates that the pre‐constructed porous structure removes gases produced during heat treatment, resulting in mechanically compressed graphene films with fewer wrinkles and high orientation. However, prolonged water immersion time (45 min) can lead to structural deterioration by causing over‐swelling of the GO film, as observed in GF‐W45, which exhibited the highest *ρ*_w_ (327 mm mm^−2^) and the lowest *f* (0.909) (Figure 4e). Surprisingly, maintaining humidity at 80, 90, and 98% enabled the construction of smaller and flatter pore structures, preventing structural damage from rapid water penetration. This resulted in a gradual decrease in *ρ*_w_ for GF‐H80, GF‐H90, and GF‐H98, from 232 to 99 mm mm^−2^, accompanied by an increase in *f* from 0.936 to 0.959 (Figure 4f–h). As depicted in Figure 4i, statistical analysis of *ρ*_w_ and *f* for graphene films prepared by different routes (Figure S3, Supporting Information) illustrates that GF‐H98 obtained after humidification treatment under 98% humidity has a flat and ordered lamellar structure, contributing to efficient phonon transport and high thermal conductivity.

### 2.4 Mechanism Analysis Based on Structure Characterizations

**Figure 5:**
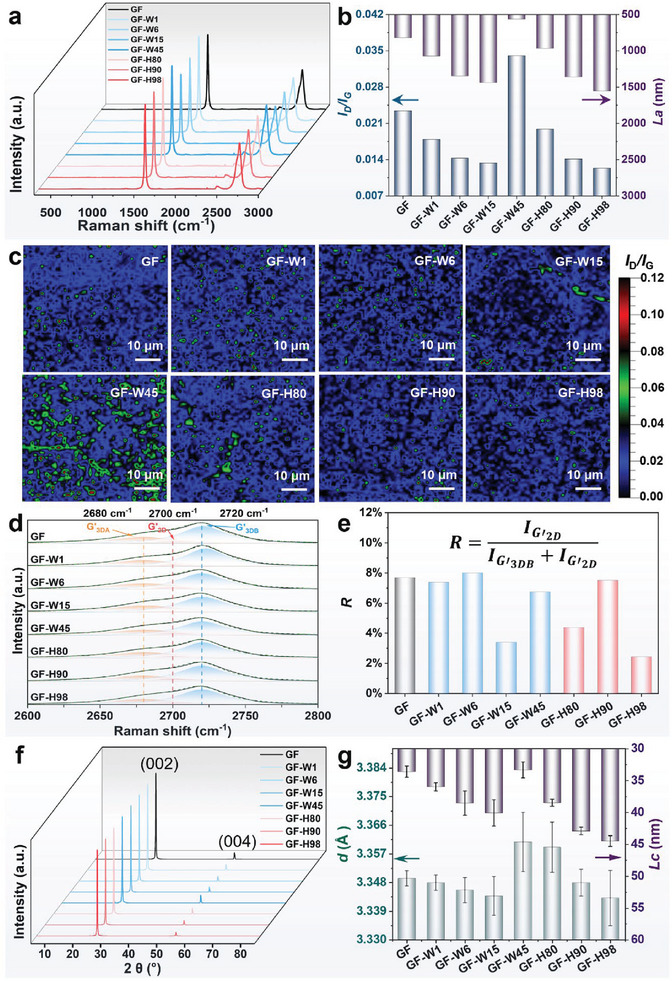
Characterization of microstructure in graphene films. a) Raman spectrum. b) Statistics of *I*_D_/*I*_G_ and *L*_a_ in graphene films. c) Raman mappings of the intensity ratio of D peak and G peak. d) Lorentzian fitting of the Raman 2D peak. e) Statistical diagram of turbostratic‐stacking proportion (*R*). f) XRD patterns. g) Statistics of average interlayer spacing (*d*) and *L*_c_ in graphene films.

The Raman spectroscopy technique utilizes the intensity ratio of the D peak to the G peak to analyze the defect quantities in graphene films.^[^ ^5^ ^]^ **Figure** **5**a shows the Raman spectra of various graphene films with a reduction of the intensity in the D peak compared with the GO film (the D and G band spectrum is shown in Figure S4, Supporting Information). Raman spectra were acquired from 2601 points within a 50 µm × 50 µm area using the Mapping mode (Section S2.4, Supporting Information). It is demonstrated that the heat annealing process effectively removes the oxygen functional groups from the GO film, restoring the *sp* ^2^ domain. However, eliminating oxygen functional groups in the form of gases (i.e., CO and CO_2_) leads to the removal of carbon atoms and disrupts the aligned sheets, leaving partial defects in the graphene film. The in‐plane grain size (*L*_a_) can be calculated using Cancado's equation,^[^ ^52^ ^]^ which is commonly employed in the study of thermally conductive graphene films.^[^ ^26^, ^36^, ^53^, ^54^ ^]^ Figure 5b shows the statistics of *I*_D_/*I*_G_ and *L*_a_ from Raman spectra of various graphene films. Compared with GF (*I*_D_/*I*_G_ value of 0.023), the *I*_D_/*I*_G_ of GF‐W15 prepared from the precursor GO film with water immersion ranging from 1 to 15 min, gradually decreased to 0.013. This indicates that the pre‐construction of porous channels within the film aids in releasing heat‐treated gases and diminishes the defects in the graphene film. However, when the water‐immersion time was extended to 45 min, the *I*_D_/*I*_G_ dramatically increased to 0.034, which was attributed to the structure disruption in the precursor GO film. It was found that ordered porous channels can be pre‐constructed in GO films at 98% humidity, resulting in a minimum *I*_D_/*I*_G_ value of 0.012 and a maximum *L*_a_ for GF‐H98. Large‐area Raman mappings of the intensity ratio of D/G demonstrate that GF‐H98 exhibits a relatively uniform and lowest *I*_D_/*I*_G_ value (Figure 5c). The G’ peak of prepared graphene films is observed at ≈2700 cm^−1^, displaying an asymmetric structure, with its intensity being sensitive to structural disorder.^[^ ^55^, ^56^ ^]^ To evaluate the stacking structure, it is assumed that it consisted of a mixture of regions with turbostratic stacking (G’_2D_) and AB Bernal stacking (G’_3DA_, G’_3DB_). The detailed Lorentzian fitting of the Raman G’ peak in Figure 5d (Figure S5, Supporting Information), reveals the fraction of turbostratic stacking (*R*) in different samples. A comparison of the *R*‐value of GF (7.7%) with those of GF‐W15 and GF‐H98, which have relatively lower *R* of 3.4% and 2.4% (Figure 5e), respectively, indicates a more ordered structure in the latter. Large‐area Raman mappings of the intensity ratio of G’_2D_/G’_3DB_ show a wide distribution of *R* values tested at different locations in the same sample. Therefore, the average value of *R* was calculated for 2601 points of the films as a reference for evaluating the stacking structure (Figure S6, Supporting Information). The grain size of graphene in the c‐axis direction (*L*_c_) is an essential parameter in reactive crystallization, as it reflects the degree of order in stacked sheets.^[^ ^55^ ^]^ By analyzing the X‐ray diffraction (XRD) pattern of the graphene films (Figure 5f; Figure S7, Supporting Information) and calculating the *L*_c_ using Scherrer's formula,^[^ ^57^ ^]^ it was determined that the average *L*_c_ of the GF‐H98 was up to 44.5 nm when the humidity was 98% (Figure 5g). This indicates that GF‐H98 exhibits optimal crystallinity and orderly sheet stacking after heat annealing. The above results confirm the benefits of pre‐constructing porous gas escape channels in GO films for preparing graphene films with low defects, high crystallinity, and high orientation. In particular, a pre‐constructed flat and ordered pore structure in the GO film facilitates the more straightforward elimination of the gas generated during heat treatment, resulting in an optimal graphene film structure with lower *ρ*_w_, *R*, *I*_D_/*I*_G_ and higher *L*_c_, *f*, which ultimately achieves an improvement in thermal conductivity (Table S3, Supporting Information).

**Figure 6:**
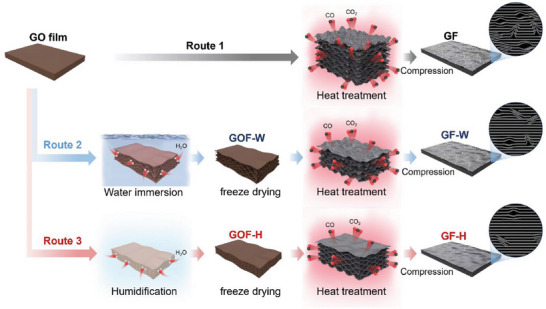
Mechanism diagram of pre‐constructed gas escape channels.

Upon heat treatment, the dense GO film exhibited substantial expansion, reaching an average expansion rate of 315% (Figure S8, Section S2.6, Supporting Information). This resulted in a wrinkled surface on the mechanically compressed GF and the formation of numerous structural defects between the film layers. The expansion occurred due to removing oxygen‐containing functional groups during the heat treatment, leading to the generation of many gases within the film. These gases were difficult to release, creating numerous gas pockets and resulting in structural defects after mechanical compression, which restricted the thermal conductivity of graphene film.^[^ ^19^, ^20^ ^]^ When the GO film was immersed in water and freeze‐dried, pre‐constructed porous channels allowed for the release of gases along these channels during heat treatment, significantly reducing film expansion to an expansion rate of only 195%. The resulting GF exhibits relatively low structural defects. However, uneven water penetration during the water immersion process disrupted interlayer pores, leading to varied sizes and misalignment. An alternative method involved treating the GO film with humidification followed by freeze‐drying, which effectively controlled water absorption, facilitated uniform water penetration between layers, and achieved an average film expansion rate of 222%. Notably, this method pre‐constructed porous channels more uniformly and orderly, aiding optimal structure in graphene film by facilitating the optimal release of gases along flat channels during heat treatment. The reduction in the expansion rate of the graphene film during the graphitization process, indicating improved integrity of the film structure, was significant when a pre‐constructed gas escape channel was in place. The GF‐W and GF‐H obtained from water immersion (Route 2) and humidification treatment (Route 3) of GO films exhibit fewer defects than GF, as depicted in **Figure** **6**.

### 2.5 Application of Heat Dissipation at High Heat Flux

**Figure 7:**
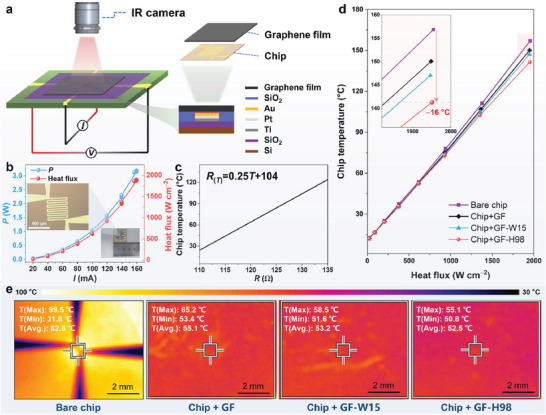
Evaluation of graphene films in heat dissipation. a) Schematic diagram of heat dissipation test platform for a chip. b) Diagram of the power on the chip as applied current changes (insert: digital photographs and optical microscope images of the chip). c) Calibration results of the linear relationship between resistance and temperature of the chip. d) Thermal performance of the samples in different heat fluxes. e) Infrared thermal imaging photographs of the chip surface.

Graphene films with high heat flux carrying capacity can effectively dissipate heat in devices with ultrahigh heat flux (10^2^–10^6^ W cm^−2^), such as multimegawatt magnetrons, fusion reactors, and synchrotron sources. To assess the heat dissipation of the samples at high heat flux, a thermal test platform was developed, consisting of a test chip, power supply, and infrared thermometer (**Figure** **7**a). Since the significant anisotropy of the graphene film and the difference between the hot spot and the film area, only the heat transfer in the in‐plane direction is concerned. The electric resistance and the applied power of the Pt‐based sensor in the test chip were measured using a four‐point probe methodology under different currents.^[^ ^21^, ^58^ ^]^ The surface area of the sensor in the chip is 400 µm × 420 µm, and the heat flux is calculated as the applied power divided by the hot spot area. The heat flux of the test chip was adjusted by varying the electric current, when a current of ≈160 mA is applied to the test chip, the heat flux reaches ≈2000 W cm^−2^ (Figure 7b). The temperature sensor in the chip was calibrated to obtain the resistance (*R*) versus the chip temperature, as illustrated in Figure 7c. Thus, the current is utilized to heat the test chip, its resistance is measured in situ, and the chip temperature is determined using the Equation (2):

(2) $$R_{T}=0.25T+104$$

where *R_T_* is the resistance of the chip under test and *T* is the chip temperature. The temperature of electronic devices is a critical factor in their performance.^[^ ^59^ ^]^ Applying a graphene film to the surface of a test chip allows the heat generated by the hotspot to be rapidly dispersed, resulting in a decrease in chip temperature. Figure 7d illustrates the temperatures of the bare chip and the chip with the affixed graphene film at varying heat flux. The results demonstrate a significant reduction in temperature for the test chip with the affixed graphene film, particularly at high heat flux. For instance, at a heat flux of ≈2000 W cm^−2^, the temperature of the bare chip is ≈157 °C but decreases to 150 °C when GF is affixed to the chip surface, resulting in a 7 °C reduction. Furthermore, affixing GF‐W15 and GF‐H98 to the test chip lowers the chip temperature by ≈10 and 16 °C, respectively. Therefore, the high thickness and thermal conductivity of graphene films lead to effective cooling of the high‐power chip. The temperature distribution on the surface of both the bare chip and the affixed samples chip, including the highest, lowest, and average temperatures, was measured using an infrared thermal imager. As shown in Figure 7e, the surface temperature distribution was captured once the test chip reached a thermally stable state. The bare chip exhibited an average surface temperature of 82.8 °C with a significant temperature difference (ΔT ≈67.7 °C). In contrast, the average surface temperatures of the bonded GF, GF‐W15, and GF‐H98 were reduced to 55.1 °C (ΔT ≈11.8 °C), 53.2 °C (ΔT ≈6.9 °C), and 52.5 °C (ΔT ≈4.3 °C), respectively. Notably, the prepared GF‐H98 demonstrated a more uniform temperature distribution and optimal heat dissipation due to its highest thermal conductivity.

## 3 Conclusions

In summary, we report a strategy for creating pathways for releasing gas generated during heat treatment to enhance the thermal conductivity of high‐thickness graphene films. By leveraging the hygroscopic expansion properties of GO films, the porous structure is constructed by controlling the introduced water content and freeze‐drying. Compared with graphene films obtained by direct heat treatment and mechanical compression of untreated GO films, immersing GO films in water and subsequently freeze‐drying them allows the formation of escape channels for gases generated during heat treatment, resulting in a 9.5% improvement in thermal conductivity of obtained graphene films. Humidification treatment of GO film with humidity enables the controlled introduction of water, leading to more uniform, interconnected, and flat pore channels. This ultimately allows for the preparation of optimal graphene films characterized by low wrinkle density, high orientation, low defects, and large grain size, resulting in an ultra‐high thermal conductivity of 1781 W m^−1^ K^−1^ at a thickness of ≈110 µm. These engineered graphene films enable effective heat dissipation, reducing the temperature by 16 °C in high heat flux devices operating at ≈2000 W cm^−2^. Our study presents a new approach for constructing porous structures in precursor GO films, overcoming the structural damage to graphene films caused by gases during heat treatment. Furthermore, this research provides valuable insights for developing new materials and technologies to enhance heat dissipation in high‐power electronic devices.

## 4 Experimental Section

### Fabrication of GO Films

The filter cake of GO (48 wt.%, Shanghai Zhongkeyueda Co. LTD.) was dispersed in deionized water (with a resistivity > 18 MΩ cm^−1^, Millipore Water Purification System) to obtain a stable dispersion of GO, followed by homogenization treatment with a high‐pressure homogenizer (JN‐30, Guangzhou Juneng Nano & Bio‐Technology Co., LTD.). The thickness of obtained GO sheets is ≈1 nm, with an average lateral size of 0.96 µm and a C/O ratio of 2.54 (Figure S9, Supporting Information). The GO films were prepared using the doctor‐blade method, a widely used technique for producing highly oriented films.^[^ ^24^, ^60^ ^]^ Initially, the GO solution (≈2 wt%) was directly spread on the polyethylene terephthalate (PET) substrates using a doctor‐blade. Subsequently, after drying at 60 °C and detaching from the PET substrates, a free‐standing GO film was obtained. Two separate routes were designed to treat the obtained GO films before heat annealing to construct gas escape channels. One route involved immersing the GOF in water for varying times (1, 6, 15, and 45 min) to achieve different degrees of water absorption, resulting in GO films (named GOF‐W1, GOF‐W6, GOF‐W15, and GOF‐W45, respectively) with porous structures after freeze‐drying. The other route entailed placing GOF in environments with different humidities (80, 90, and 98%) to absorb moisture to saturation (Section S1, Supporting Information), leading to films (named GOF‐H80, GOF‐H90, and GOF‐H98, respectively) with porous structures after freeze‐drying (Figure S2, Supporting Information).

### Preparation of Graphene Films

To prevent films from expanding uncontrollably during subsequent heat annealing, the GO films from earlier were pre‐treated at 80–205 °C for 12 h to eliminate residual water and partial oxygen functional groups.^[^ ^23^, ^61^ ^]^ Afterward, the pre‐treated GO films were heated up to 3150 °C for 2 h in a furnace under flowing argon, to form expanded graphene films. Finally, several expanded graphene films were stacked and mechanically pressed under 5 MPa and a rolling speed of 0.1 mm^−1^s^−1^ using a dynamic rolling presser to fabricate the highly densified and thick graphene films (> 2.15 g cm^−3^, ≈110 µm). The density and porosity of graphene films are shown in Table S4 (Supporting Information). The GO films were obtained through different methods were then exposed to heat annealing and mechanical compression to produce graphene films designated as GF, GF‐W1, GF‐W6, GF‐W15, GF‐W45, GF‐H80, GF‐H90, and GF‐H98, respectively.

### Heat Dissipation Performance Measurement

To evaluate the heat dissipation and cooling effect of the as‐prepared graphene films on the chip, a thermal test platform was built. The platform featured a platinum‐based chip as the heat source, a DC power supply for controlling the heat flux, and an infrared thermal imaging camera for analyzing the surface temperature distribution of the samples. The chip consisted of silicon (Si), silicon oxide (SiO_2_), titanium (Ti), platinum (Pt), and gold (Au). Specifically, a Si wafer and SiO_2_ were employed as the substrate, and a Ti/Pt/Au film was evaporated after the circuit pattern is transferred to the substrate, followed by the sputtering of a SiO_2_ insulating layer on the chip.^[^ ^21^, ^58^ ^]^ The resistance and applied power of the chip were measured using the four‐point probe method and the heat flux through the chip at ≈2000 W cm^−2^ by adjusting the electric current from the DC power supply. Graphene films with a size of 12.5 mm were tightly attached to the chip surface to aid in heat dissipation. The temperature variations of the chip with various samples were recorded, and the surface temperature distribution was studied. Finally, the heat dissipation and cooling effects of the samples on the chip were comprehensively evaluated.

### Structural Analysis and Characterization

The detailed characterization methods are provided in Supporting Information.

## Conflict of Interest

The authors declare no conflict of interest.

## Supporting information



Supporting Information

## Data Availability

The data that support the findings of this study are available in the supplementary material of this article.
