# Supplementary material for: Achieving Ultra‐High Heat Flux Transfer in Graphene Films via Tunable Gas Escape Channels
Source: Adv Sci (Weinh). 2024 Nov 11;12(1):2410913. doi: 10.1002/advs.202410913 (PMC11714209; doi:10.1002/advs.202410913)
Supplement: Supplementary file 1 — Supporting Information [file ADVS-12-2410913-s001.pdf]

ADVANCED SCIENCE
Open Access

Supporting Information

for *Adv. Sci.*, DOI 10.1002/advs.202410913

Achieving Ultra-High Heat Flux Transfer in Graphene Films via Tunable Gas Escape Channels

Haolong Zheng, Peng He, Shujing Yang, Yonghua Lu, Na Guo, Yanhong Li, Gang Wang* and Guqiao Ding**

Supporting Information

Achieving Ultra-High Heat Flux Transfer in Graphene Films via Tunable Gas Escape Channels

Haolong Zheng, *Peng He**, *Shujing Yang*, *Yonghua Lu*, *Na Guo*, *Yanhong Li*, *Gang Wang**, *Guqiao Ding**

H. Zheng, P. He, S. Yang, Y. Lu, N. Guo, Y. Li, G. Wang, G. Ding
State Key Laboratory of Materials for Integrated Circuits, Shanghai Institute of Microsystem and Information Technology, Chinese Academy of Sciences, Shanghai 200050, P. R. China
E-mail: hepeng@mail.sim.ac.cn; gangwang@nbu.edu.cn; gqding@mail.sim.ac.cn

H. Zheng, P. He, S. Yang, G. Ding
College of Materials Science and Opto-Electronic Technology, University of Chinese Academy of Sciences, Beijing 100049, P. R. China

Y. Lu, N. Guo, Y. Li
Zhongke Yueda Shanghai Material Technology Co., Ltd, Shanghai 201800, P. R. China

G. Wang
School of Physical Science and Technology, Ningbo University, Ningbo 315211, P. R. China

Contents

S1. Fabrication of GO Films
S2. Characterization Section
S3 Supplementary Figures
S4 Supplementary Tables

S1 Fabrication of GO Films

The dried GO films prepared using a blade coater (CHTB-04) were cut into 8 cm × 8 cm pieces, and then immersed in deionized water. By adjusting the immersion time from 1 min to 45 min, the degree of water absorption and swelling of the GO film increased over time. Subsequently, the hydrous and swollen GO films were subjected to rapid freeze-drying using a lyophilizer (SCIENTZ-18N) to obtain GOF-W1, GOF-W6, GOF-W15, and GOF-W45. Furthermore, the cut GO films were placed in a constant temperature and humidity test chamber (DHTHM-50-0-P-SD) set at 25 °C with 80%, 90%, and 98% humidity for 3 h until the films were saturated with moisture absorption and exhibited no further weight change. Following this, the GO films were freeze-dried as previously described to obtain GOF-H80, GOF-H90, and GOF-H98. The dried GO film without any treatment was freeze-dried and named as GOF-D. The weight, thickness, and density of different GO films were compared as shown in **Figure S1**. The freezing process of dried GO film with no free water cannot form ice crystals. Therefore, freeze-drying does not have a significant effect on the macrostructure (thickness, density) of the already-dried GO film.

S2 Characterization Section

S2.1 Morphological and Structural Characterization of GO Sheets

The lateral size and thickness of GO sheet were analyzed using scanning electron microscopy (SEM, Sigma 300) and atomic force microscopy (AFM, Bruker Dimension Edge) were used to analyze, as shown in **Figure S9**. The GO dispersion was deposited onto SiO_2 substrates for SEM and AFM characterization. Furthermore, the elemental composition of the GO feedstock and the assignment of carbon peaks were determined using X-ray photoelectron spectroscopy (XPS, 250 Xi).

S2.2 Measurement of Thermal Conductivity

The thermal conductivity of the graphene films was evaluated using laser flash analysis (NETZSCH LFA 467). The samples were cut into 25.4 mm diameter discs and placed in in-plane holders for testing at 25°C. During the measurement, a laser source emitted light pulses to instantaneously heat the center of the lower surface, creating a point source of heat. The generated heat then propagates through the sample to the upper surface, where an infrared

detector recorded the temperature rise. With the recorded temperature data, the thermal diffusivity (α) of the sample can be determined using the following equation (S1):

$$\alpha = 0.1388 \cdot \frac{d}{t_{50}}^2, \quad \text{(S1)}$$

where d represents the thickness of the sample, and t_{50} indicates the half-heating time, which is the duration it takes for upper surface of the sample to reach half of its maximum value after being exposed to a light pulse. The thermal conductivity (k) of the sample can be calculated using the following equation (S2):

$$k = \alpha \cdot C_p \cdot \rho \,, \quad \text{(S2)}$$

where $\boldsymbol{C_p}$ represents the specific heat of the sample and $\boldsymbol{\rho}$ represents the sample's density.[1–6] The average thickness of the graphene films was obtained by measuring different film positions using a thickness gauge (Mitutoyo 547-400s).

S2.3 Characterization of GO Film Structure

X-ray diffraction (XRD) analyses were conducted using a Bruker D8 ADVANCE X-ray diffractometer equipped with a monochromatic source of Cu $K\alpha 1$ radiation. The XRD was used to characterize the GO films prepared through different routes to determine the variation in layer spacing. The layer spacing (d_L) of GO films was calculated using the Bragg's equation (S3) based on the XRD (002) peak:

$$n\lambda = 2d \cdot \sin\theta, \quad \text{(S3)}$$

where λ is the X-ray wavelength (0.15406 nm), and θ (rad) is the scattering angle.

The pore structure inside GO film was analyzed using three-dimensional X-ray diffraction microscopy (XRM, ZEISS Xradia 510 Versa). Each sample underwent a 5 h scan at 140 kV voltage and 10 W power to acquire 2001 cross-sectional images. These images were processed using Dragonfly Pro software to generate 3D reconstructed photographs and pore information. The reconstructed cross-section images were further analyzed using Fourier Fast Transform (FFT) in Image J to analyze the distribution of the pores, there the complex spatial patterns represented by gray values in each pixel $I[m,n]$ of an image is converted into direction dependent frequency components ($F[u,v]$), as the following equation (S4):[7]

$$F[u,v] = \frac{1}{MN}\sum_{m=0}^{M-1}\sum_{n=0}^{N-1} I[m,n] exp\left(-i2\pi\left(\frac{um}{M} + \frac{vn}{N}\right)\right), \quad \text{(S4)}$$

The intensity of each pixel is influenced by the spatial alignment patterns, with high-intensity pixels clustering along the orientation with the highest degree of directional anisotropy. The FFT method is commonly used for analyzing films orientation.[6,8,9] In FFT images of

reconstructed cross-section, the angular distribution of the intensity in the frequency domain images fits well demonstrated a good fit using the Cauchy–Lorentz distributions (S5):

$$y = y_0 + \frac{2A}{\pi}\left(\frac{\omega}{4(x-x_0)^2+\omega^2}\right), \quad \text{(S5)}$$

The position parameter (x_0) of the distribution indicates the angle corresponding to the peak of the curve, which is approximately 90° for the orientation along the in-plane of film. The scale parameter (ω) measures the alignment degree by specifying the half width at half maximum (HWHM), indicating the angle of deviation from the principal direction. Therefore, lower HWHM values signify better alignment.

S2.4 Characterization of Graphene Film Morphology and Structure

SEM at 5 kV was utilized to analyze the morphology of the graphene film surface. The obtained images were then analyzed using Image J to determine the wrinkle density of the film surface (ρ_w) using the following equation (S6):

$$\rho_w = \frac{l_w}{S}, \quad \text{(S6)}$$

where l_w is the total length of the wrinkles, and S is the area of the image region. This method is suitable for the quantitative analysis of wrinkles on flatter film surfaces.[6,10,11]

Wide-angle X-ray scattering (WAXS) has been extensively employed to assess the orientation of thin films assembled from two-dimensional sheets.[9,12–15] WAXS measurements were performed on a Nano-star SAXS System using an incident Cu-Kα X-ray beam that is parallel to the film plane. The samples were cut into 1 mm × 10 mm rectangular strips, and the distance between the sample and the detector was 60 mm during the WAXS measurement. The scattering patterns were collected by the Vantec 2000 detector. The orientation of graphene sheets in films was quantified using the (002) reflection in WAXS patterns. The orientation was quantified by converting the orientation distribution into a Herman's order parameter (*f*), defined by equation (S7):

$$f = \frac{1}{2}(3\langle cos^2 \phi\rangle - 1), \quad \text{(S7)}$$

where $\langle cos^2 \phi\rangle$ is the average value of the square of the cosine of the azimuthal angle ϕ for the 002 peak of graphene films, calculated as follows in equation (S8):

$$\langle cos^2 \phi\rangle = \frac{\int_0^{\pi/2} I(\phi)\, cos^2 \phi\, sin\, \phi d\phi}{\int_0^{\pi/2} I(\phi)\, sin\, \phi d\phi}, \quad \text{(S8)}$$

where $I(\phi)$ is the intensity at an azimuthal angle of ϕ. The intensity is normalized according to equation (S9):

$$\int_0^{\pi/2} I(\phi)\, sin\, \phi\, d\phi = 1, \tag{S9}$$

The intensity of WAXS distribution of the graphene films was well-represented using the Gauss distributions (S10):

$$y = y_0 + A * exp\left(-\frac{1}{2}(\frac{x-x_c}{w})^2\right), \tag{S10}$$

where the w is the full width of half maxima (FWHM), representing the azimuthal angle distribution.

Raman spectroscopy (Renishaw Micro-Raman) was used to examine the defects (I_D/I_G), grain size in plane (L_a), and proportion of turbostratic stacking (R) of graphene films. Raman spectra were acquired in mapping mode using a laser with a wavelength of 532 nm. Raman mapping conducted within a 50 μm × 50 μm area on the sample surface with a fitting step. The average value of I_D/I_G was statistically derived from the intensity ratio of D peak and G peak of Raman mappings (2601 point), while L_a was calculated by following equation (S11):[16]

$$L_a = (2.4 \times 10^{-10}) \times \lambda_l^4 \times (\frac{I_D}{I_G})^{-1}, \tag{S11}$$

where λ_l represents laser wavelength. Additionally, the Raman G' peak indicated a mixture of turbostratic stacking and AB Bernal stacking structure in the graphene films. To evaluate the relative volume of turbostratic stacking, the G' peak was fitted by three Lorentzian components (**Figure S5**), which included G'_{2D} (2700 cm^{-1}) corresponding to turbostratic stacking, G'_{3DA} (2680 cm^{-1}) and G'_{3DB} (2720 cm^{-1}) corresponding to AB Bernal stacking. The relative volume of turbostratic (R) in graphene films was calculated based on the following equation (S12):[17,18]

$$R = \frac{I_{G'_{2D}}}{I_{G'_{2D}} + I_{G'_{3DB}}}, \tag{S12}$$

The average value of R was derived from the Raman mappings (2601 point) of graphene films (**Figure S6**).

XRD (Bruker D8 ADVANCE) was employed to analyze the grain size in C-axis (L_c) of graphene films. The layer spacing (d_L) of the graphene film was determined using Bragg's equation (S3). Scherrer equation (S13) was utilized to estimate L_c.[19,20]

$$L_c = \frac{k\lambda}{\beta \cos\theta}, \tag{S13}$$

where k is a constant (0.89), λ is the X-ray wavelength (0.15406 nm), θ is the scattering angle, and β is the full width at half maximum (FWHM) of the (002) peak.

S2.5 Thickness measurement of GO films

The thickness of GO films was used a thickness gauge (Mitutoyo 547-400s) in different points (13 points) of the single film with an area of 10 cm × 10 cm. (**Figure S10**a). The average thickness statistics of GO films are shown in **Figure S10**b, the error bars indicate that the GOF-W series films have a higher thickness deviation compared to that of GOF-H. As shown in **Figure S10**c, the thickness decreases the closer it is to the central region of GO films, especially for the GOF-W samples.

S2.6 The measurement of film expansion rate

The change in film thickness is a key parameter to evaluate the evolution of the film structure. Since the thickness of the precursor graphene oxide films is different, it is not appropriate to compare the thickness changes of different samples directly. Therefore, we calculated the expansion rate before and after the treatment of different films. The expansion rate (E) is given by the formula: $E = \frac{T_2 - T_1}{T_1} \times 100\%$, where T_1 is the thickness of the untreated film and T_2 is the thickness of the treated film. The average thickness of the films was obtained by measuring different film positions using a thickness gauge (Mitutoyo 547-400s). As shown in **Figure S8**, we calculated the expansion rate (E_1, gray) of the initial GO film before and after pretreatment (water immersion or humidification treatment), the expansion rate (E_2, orange) of the pre-treated GO film before and after graphitization, and the expansion rate (E_3, red) of the initial GO film before and after final graphitization. It can be found that the pre-constructed gas channel by water immersion or humidification treatment increases E_1, which is conducive to the release of gases during heat treatment and finally reduces E_3.

S3 Supplementary Figures

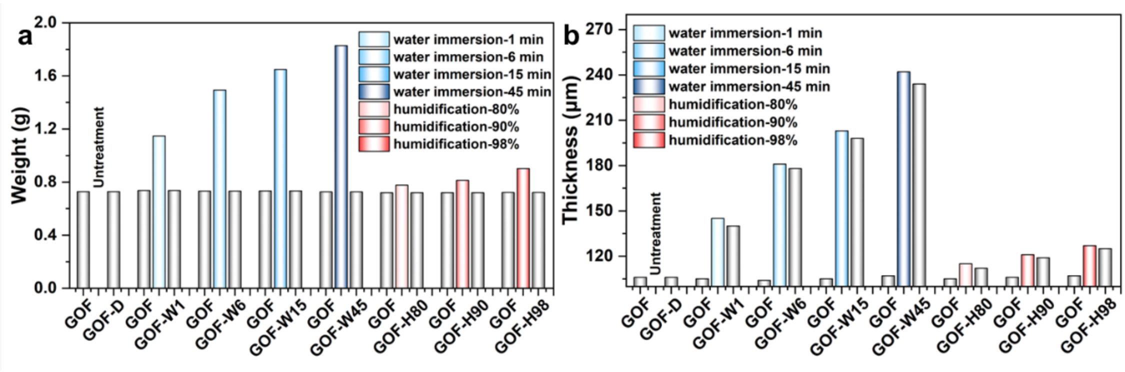

Figure S1. Changes of weight and thickness in GO film by different routes. a) The weight change of GO films. b) The thickness change of GO films.

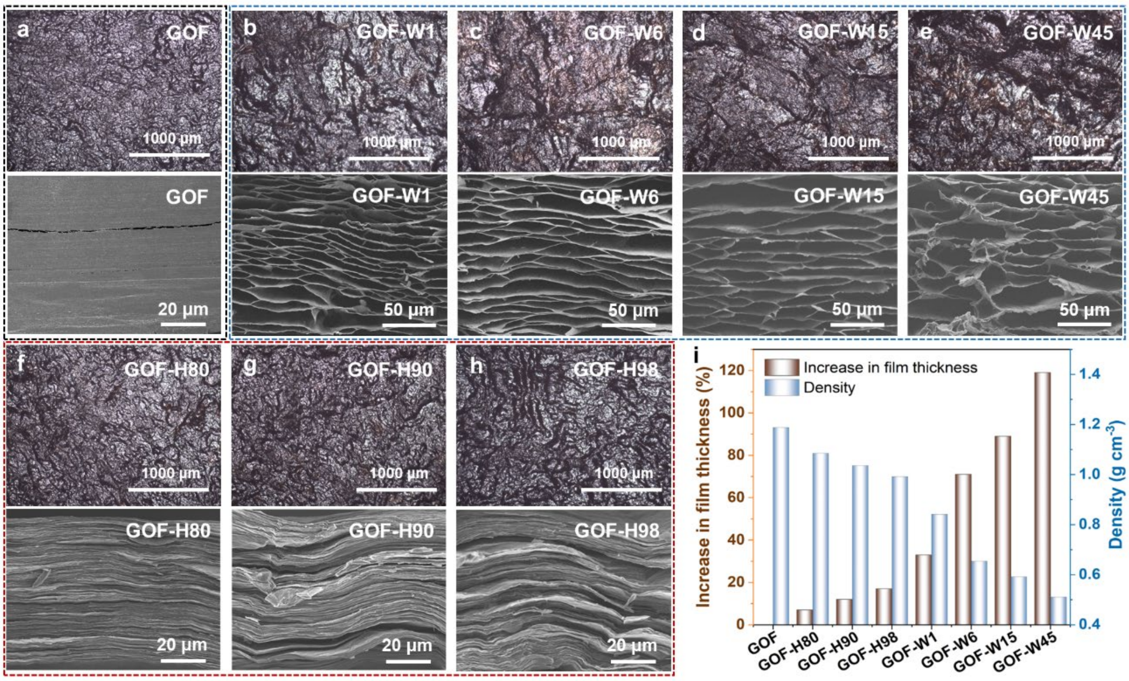

Figure S2. Surface photograph (optical microscopy) and cross-section images (SEM) of a) GOF, b) GOF-W1, c) GOF-W6, d) GOF-W15, e) GOF-W45, f) GOF-H80, g) GOF-H90, and h) GOF-H98. i) Thickness increase and density of different GOF.

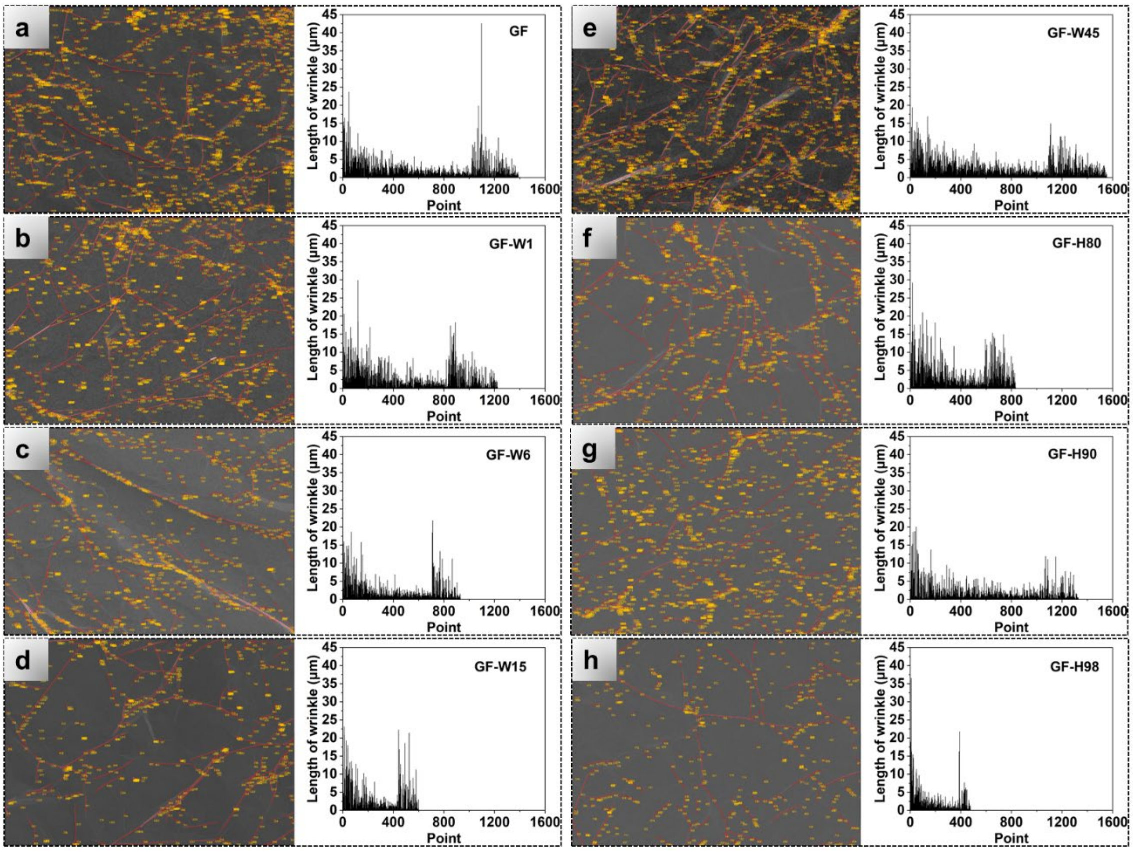

Figure S3. Distribution and length statistics (the red lines are wrinkles, and the yellow numbers are statistical wrinkles) of wrinkles on the surface of a) GF, b) GF-W1, c) GF-W6, d) GF-W15, e) GF-W45, f) GF-H80, g) GF-H90, and h) GF-H98.

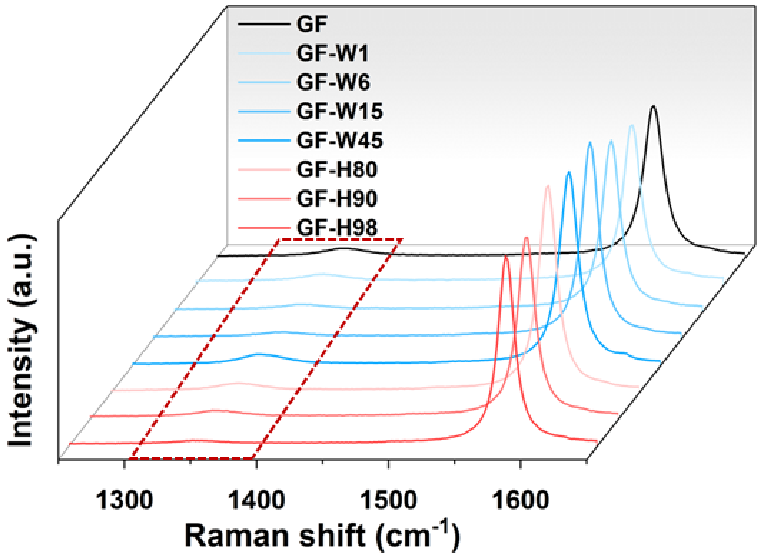

Figure S4. Raman spectra of graphene films in the 1250 cm^{-1} to 1650 cm^{-1} band.

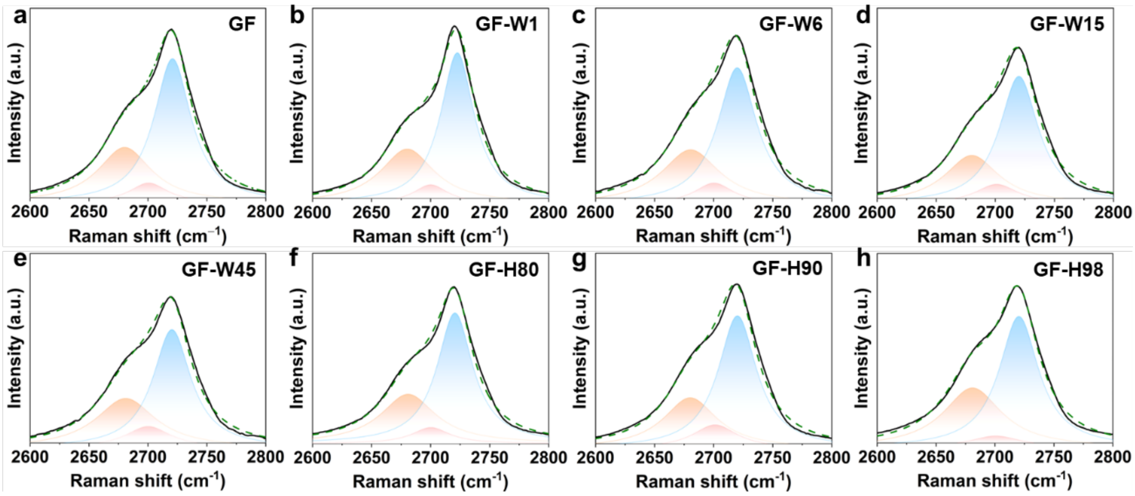

Figure S5. Lorentzian fitting of the Raman G' peak of a) GF, b) GF-W1, c) GF-W6, d) GF-W15, e) GF-W45, f) GF-H80, g) GF-H90, and h) GF-H98.

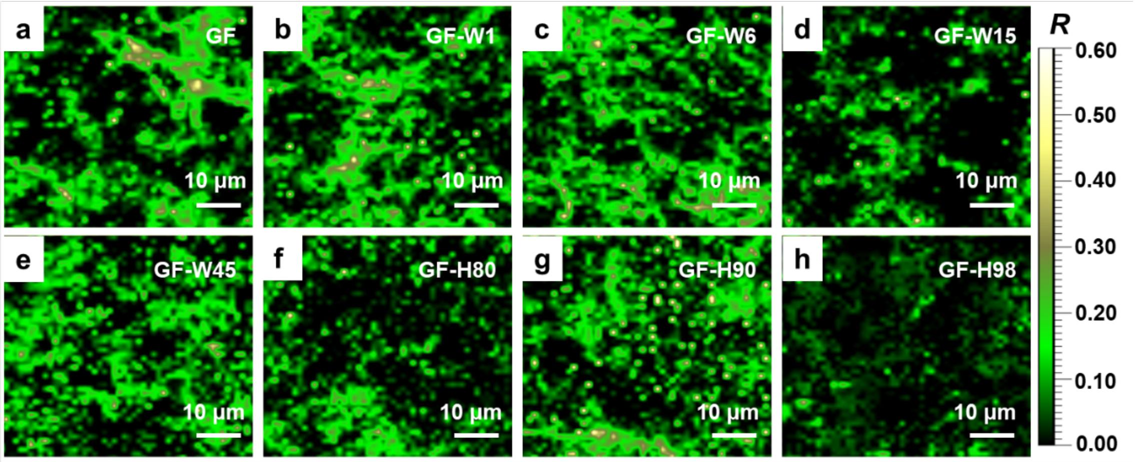

Figure S6. Evaluation of the proportion of turbostratic-stacked regions in graphene films. Raman mappings of the intensity ratio of $G'_{2D}/(G'_{2D}+G'_{3DB})$ of a) GF, b) GF-W1, c) GF-W6, d) GF-W15, e) GF-W45, f) GF-H80, g) GF-H90, and h) GF-H98.

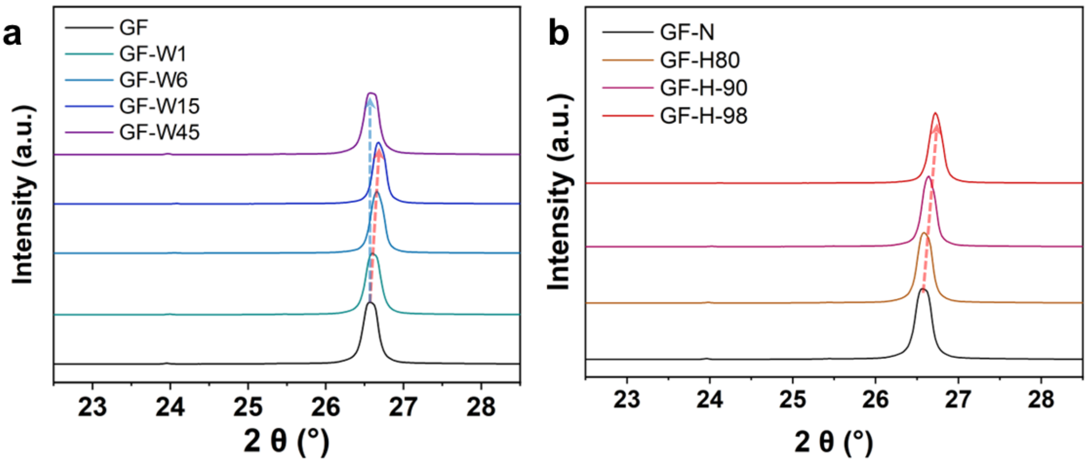

Figure S7. XRD patterns. a) Comparison on zoomed-in XRD patterns of samples prepared by route 1 and Route 2. b) Comparison on zoomed-in XRD patterns of samples prepared by route 1 and Route 3.

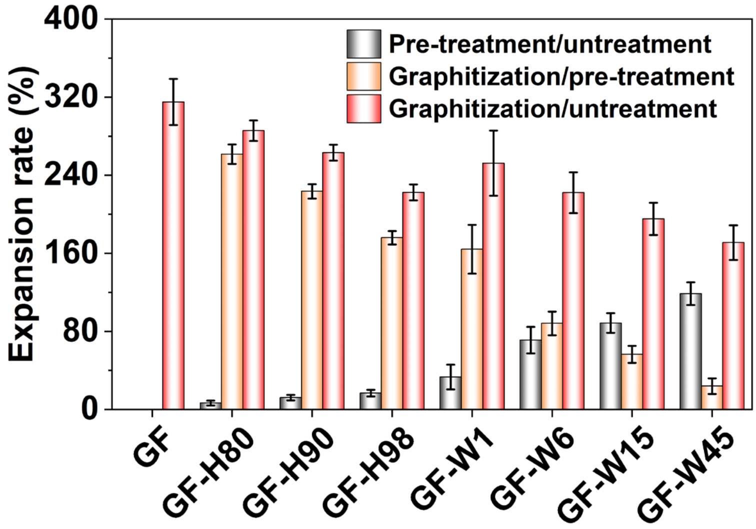

Figure S8. Evaluation of film expansion.

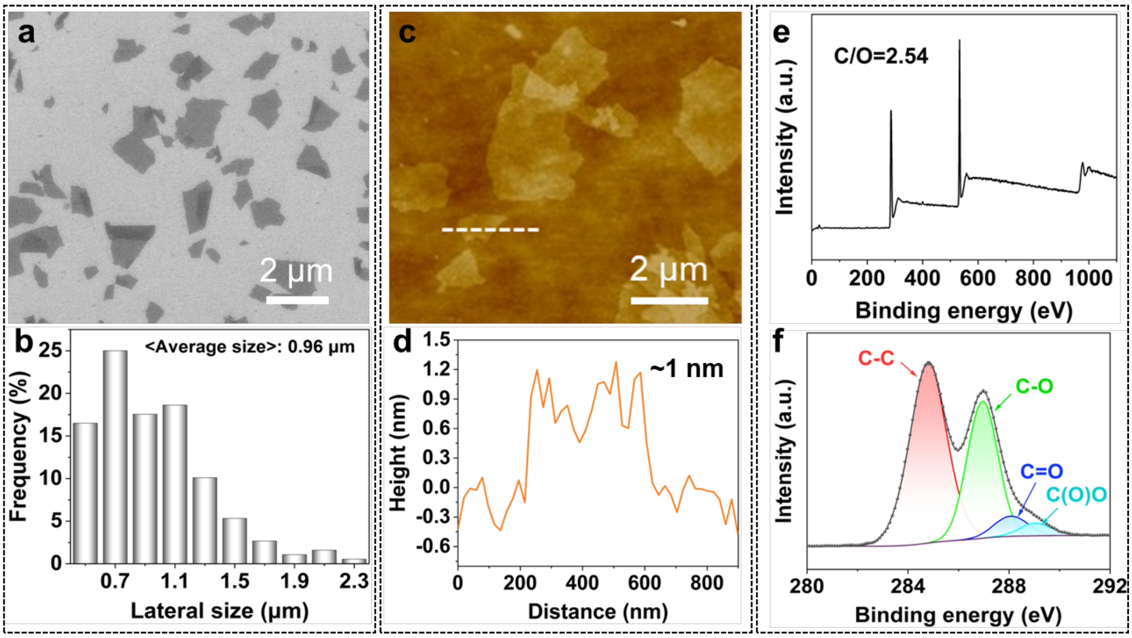

Figure S9. Morphological and structural characterization of GO sheets. a)-b) Typical SEM image and statistics in the lateral size of GO sheets. c)-d) Typical AFM image and thickness analysis of GO sheets. e)-f) XPS spectra and high-resolution XPS (C 1s) analysis of GO.

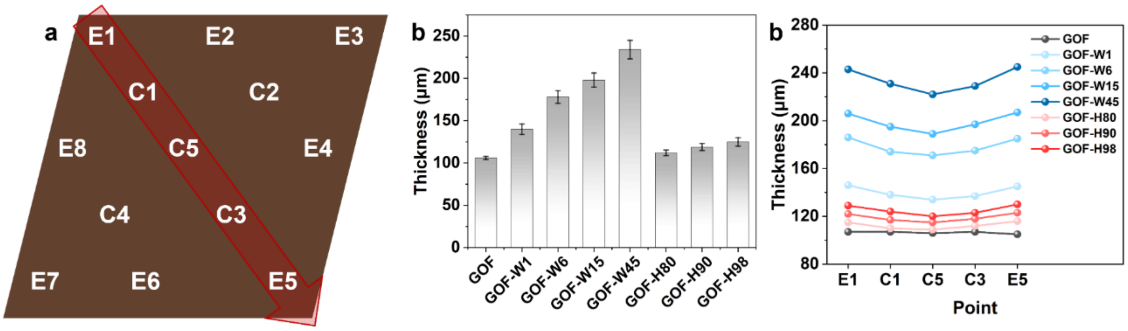

Figure S10. Evaluation of uniformity in GO film thickness. a) Schematic diagram of the thickness measurement site in square GO film. b) Histogram of the thickness distribution of GO films. c) Distribution of thickness in diagonal point of square GO film.

S4 Supplementary Tables

Table S1. Comparison of the thermally conductive graphene films fabricated from different methods.

Materials	Method	Heat treatment (°C)	*d* (μm)	*k* (W m^{-1} K^{-1})	$d \times k$ (W K^{-1})	Year	Ref.
GO	Blade-coating	3150	110 ± 2<br>140 ± 3<br>190 ± 5<br>220 ± 6	1781 ± 25<br>1735 ± 13<br>1685 ± 8<br>1639 ± 19	0.1959<br>0.2429<br>0.3201<br>0.3605	This year	**Our work**
GO/GNP[a)]/PDA[b)]	Blade-coating	2800	25	1300	0.0325	2024	[21]
GO	Blade-coating	3150	86	1629	0.1400	2024	[9]
GO	Blade-coating	3150	110	1550	0.1705	2024	[6]
GO	EISA[c)]	2850	1	3826	0.0038	2023	[22]
N-rGO[d)]	Filtration	3000	10	1700	0.0170	2023	[20]
GO/CNT	Filtration	2800	140	1038	0.1453	2023	[23]
GO/Fe-CNC	Blade-coating	1500	7.8	1958	0.0152	2023	[24]
GO/PAN[e)]	Roll-coating	3000	10	1581	0.0158	2023	[11]
GO/CNT	Blade-coating	2800	30	1208.3	0.0362	2023	[25]
GO/VC	Blade-coating	2800	680	1042	0.7085	2023	[26]
GO	Spin coating	2800	20<br>100	1565<br>1265	0.0313<br>0.1265	2022	[27]
GO	Blade-coating	2850	100	1576	0.1576	2022	[28]
GNP[a)]	Roll-coating	2800	80	950.31	0.0760	2021	[29]
GO	Filtration	3000	0.024	2820	0.000067	2021	[12]
GNP[a)]	Filtration	600	65	975	0.0633	2021	[30]
GO/PNA[f)]	Filtration	2800	5	1016.3	0.0050	2021	[31]
GO/PAN[e)]	Blade-coating	2800	3	1282	0.0038	2021	[32]
GO	Blade-coating	2800	25	1314	0.0328	2021	[33]
GO/VC	Blade-coating	2950	80	1600	0.1280	2021	[34]
GO	Wet spinning	NA	8.4	1102	0.0092	2020	[35]
GO/VC	EISA[c)]	800	15	1031.9	0.01547	2020	[36]
GO/PyM[g)]	EISA[c)]	3000	3.5	1316	0.0046	2020	[37]
GO/PI[h)]	Hot-pressing	2800	15	1427	0.0214	2020	[38]
GO/rGO	Blade-coating	3000	2	2025	0.0040	2020	[39]

GO	Blade-coating	2850	75<br>220	1204<br>1070	0.0903<br>0.2354	2020	[40]
GO	Blade-coating	2800	200	1224	0.2448	2020	[41]
GO/PI[h)]	Blade-coating	2300	30	1428	0.0428	2020	[42]
GO	EISA[c)]	1500	2	1352	0.0027	2019	[1]
GO/Glu[i)]	Filtration	1000	15	1331	0.0199	2019	[43]
GO/PDA[b)]	Filtration	3000	10	1584	0.0158	2019	[44]
GtO/PAN[e)]	Blade-coating	2850	20	1629	0.0325	2019	[45]
GO	Dry-bubbling	2850	0.8	3200	0.0025	2018	[17]
GO	Hot-pressing	2000	80	1103	0.0882	2018	[46]
GO/CNC	EISA[c)]	1500	36	1820.4	0.0655	2018	[47]
GO/PI[h)]	EISA[c)]	2800	3	1269.7	0.0038	2018	[48]
GO	Blade-coating	2750	20	1200	0.0240	2018	[49]
GNP[a)]	Filtration	2850	30	1529	0.0458	2017	[50]
GNP[a)]	Filtration	2800	15	1324	0.0198	2017	[51]
GNP[a)]	Filtration	2800	10	1842	0.0184	2017	[52]
GO	Blade-coating	3000	10	1940	0.0194	2017	[5]
GO	Filtration	NA	15	1390	0.0208	2015	[53]
GO	Filtration	NA	20<br>40<br>60	1642<br>1234<br>675	0.0328<br>0.0493<br>0.0405	2015	[54]
rGO	ESD[j)]	2850	25	1434	0.0358	2014	[55]
GO	EISA[c)]	2000	8.4	1100	0.0092	2014	[56]

[a)] Graphene nanoplates; [b)] Polydopamine; [c)] Evaporation-induced self-assembly; [d)] Nitrogen doped-reduced GO; [e)] Polyacrylonitrile; [f)] Poly-naphthylamine; [g)] 1-pyrenemethanol; [h)] Polyimide; [i)] Glucose [j)] Electron spray deposition.

Table S2. Comparison of 2θ position, layer spacing, and film expansion rate of prepared GO films.

Samples	2θ position (°)	Layer spacing, d_L (nm)	Film expansion rate (%)
GOF	12.695	0.697	0
GOF-H80	11.487	0.770	7
GOF-H90	11.273	0.784	12
GOF-H98	11.039	0.801	17
GOF-W1	9.869	0.896	33
GOF-W6	8.136	1.086	71
GOF-W15	7.065	1.250	89
GOF-W45	6.948	1.271	119

Table S3. Comparison of average d_L, L_c, I_D/I_G, L_a, R, ρ_w, and f of prepared graphene films.

Samples	Layer spacing, d_L (nm)	L_c (nm)	I_D/I_G	L_a (nm)	R (%)	ρ_w (mm mm^{-2})	f
GF	0.3349±0.002	33.60±0.9	0.0234	820.86	7.70	258	0.91
GF-W1	0.3347±0.002	35.99±0.6	0.0179	1072.71	7.39	255	0.913
GF-W6	0.3345±0.004	38.56±1.9	0.0143	1346.79	8.00	165	0.942
GF-W15	0.3343±0.006	40.09±2.08	0.0134	1439.39	3.41	139	0.951
GF-W45	0.3361±0.009	33.33±1.2	0.0340	564.72	6.76	327	0.909
GF-H80	0.3359±0.007	38.50±0.5	0.0199	967.27	4.38	232	0.936
GF-H90	0.3348±0.004	42.94±0.6	0.0141	1360.08	7.52	194	0.946
GF-H98	0.3343±0.008	44.53±0.8	0.0124	1553.99	2.44	99	0.959

Table S4. Statistical of density and porosity of graphene films.

Sample	Density (g cm^{-3})	Porosity (%)
GF	2.219	2.15
GF-W1	2.220	2.10
GF-W6	2.222	2.01
GF-W15	2.225	1.86
GF-W45	2.218	2.17
GF-H80	2.221	2.06
GF-H90	2.222	1.98
GF-H98	2.225	1.86

References

[1] X. Wu, H. Li, K. Cheng, H. Qiu, J. Yang, *Nanoscale* **2019**, *11*, 8219.

[2] D. Hu, W. Gong, J. Di, D. Li, R. Li, W. Lu, B. Gu, B. Sun, Q. Li, *Carbon* **2017**, *118*, 659.

[3] S. Jin, Q. Gao, X. Zeng, R. Zhang, K. Liu, X. Shao, M. Jin, *Diam. Relat. Mater.* **2015**, *58*, 54.

[4] J. Zhang, G. Shi, C. Jiang, S. Ju, D. Jiang, *Small* **2015**, *11*, 6197.

[5] L. Peng, Z. Xu, Z. Liu, Y. Guo, P. Li, C. Gao, *Adv. Mater.* **2017**, *29*, 1700589.

[6] S. Yang, P. He, H. Zheng, D. Xiao, G. Ding, *Chem. Eng. J.* **2024**, *493*, 152803.

[7] X. Lin, X. Shen, Q. Zheng, N. Yousefi, L. Ye, Y. W. Mai, J. K. Kim, *ACS Nano* **2012**, *6*, 10708.

[8] J. Chen, P. He, T. Huang, D. Zhang, G. Wang, S. Yang, X. Xie, G. Ding, *Nano Energy* **2021**, *90*, 106593.

[9] Y. Quan, P. He, J. Chen, N. Guo, Y. Li, H. Zheng, J. Zhang, X. Ren, Y. Zhang, W. Bao, K. Qi, G. Ding, *Carbon* **2024**, *226*, 119179.

[10] J. Lin, P. Li, Y. Liu, Z. Wang, Y. Wang, X. Ming, C. Gao, Z. Xu, *ACS Nano* **2021**, *15*, 4824.

[11] S. Luo, L. Peng, Y. Xie, X. Cao, X. Wang, X. Liu, T. Chen, Z. Han, P. Fan, H. Sun, Y. Shen, F. Guo, Y. Xia, K. Li, X. Ming, C. Gao, *Nano-Micro Lett.* **2023**, *15*, 61.

[12] L. Peng, Y. Han, M. Wang, X. Cao, J. Gao, Y. Liu, X. Chen, B. Wang, B. Wang, C. Zhu, X. Wang, K. Cao, M. Huang, B. V. Cunning, J. Pang, W. Xu, Y. Ying, Z. Xu, W. Fang, Y. Lu, R. S. Ruoff, C. Gao, *Adv. Mater.* **2021**, 2104195.

[13] M. Tan, D. Chen, Y. Cheng, H. Sun, G. Chen, S. Dong, G. Zhao, B. Sun, S. Wu, W. Zhang, J. Han, W. Han, X. Zhang, *Adv. Funct. Mater.* **2022**, 2202057.

[14] S. Wan, Y. Chen, S. Fang, S. Wang, Z. Xu, L. Jiang, R. H. Baughman, Q. Cheng, *Nat. Mater.* **2021**, *20*, 624.

[15] P. Li, M. Yang, Y. Liu, H. Qin, J. Liu, Z. Xu, Y. Liu, F. Meng, J. Lin, F. Wang, C. Gao, *Nat. Commun.* **2020**, *11*, 2645.

[16] L. G. Cançado, K. Takai, T. Enoki, M. Endo, Y. A. Kim, H. Mizusaki, A. Jorio, L. N. Coelho, R. Magalhães-Paniago, M. A. Pimenta, *Appl. Phys. Lett.* **2006**, *88*, 163106.

[17] N. Wang, M. K. Samani, H. Li, L. Dong, Z. Zhang, P. Su, S. Chen, J. Chen, S. Huang, G. Yuan, X. Xu, B. Li, K. Leifer, L. Ye, J. Liu, *Small* **2018**, *14*, 1801346.

[18] L. G. Cançado, K. Takai, T. Enoki, M. Endo, Y. A. Kim, H. Mizusaki, N. L. Speziali, A. Jorio, M. A. Pimenta, *Carbon* **2008**, *46*, 272.

[19] M. S. Seehra, A. S. Pavlovic, *Carbon* **1993**, *31*, 557.

[20] Q. Zhang, Q. Wei, K. Huang, Z. Liu, W. Ma, Z. Zhang, Y. Zhang, H. Cheng, W. Ren, *Natl. Sci. Rev.* **2023**, *10*, 147.

[21] K. Xiong, T. Yang, Z. Sun, C. Ma, J. Wang, X. Ge, W. Qiao, L. Ling, *Carbon* **2024**, *219*, 118827.

[22] S. Guo, S. Chen, A. Nkansah, A. Zehri, M. Murugesan, Y. Zhang, Y. Zhang, C. Yu, Y. Fu, M. Enmark, J. Chen, X. Wu, W. Yu, J. Liu, *2D Mater.* **2023**, *10*, 014002.

[23] W. Xu, A. G. Olatoye, Y. Cui, *J. Mater. Sci.* **2023**, *58*, 9502.

[24] J. Zhang, X. Zhang, J. Shen, H. Pan, Z. Chen, Y. Li, S. Zhu, *Carbon* **2023**, *201*, 295.

[25] K. Xiong, C. Ma, J. Wang, X. Ge, W. Qiao, L. Ling, *Ceram. Int.* **2023**, *49*, 8847.

[26] S. Yang, Z. Tao, Q. Kong, J. Li, X. Li, X. Yan, J. Liu, Y. Tong, Z. Liu, *Chem. Eng. J.* **2023**, *473*, 145330.

[27] D. Liu, H. Fu, T. Yang, W. Wang, J. Zhao, K. Wu, C. Wu, Z. Yong, Y. Zhang, *Mater. Res. Express.* **2022**, *9*, 036405.

[28] P. Zhang, P. He, Y. Zhao, S. Yang, Q. Yu, X. Xie, G. Ding, *Adv. Funct. Mater.* **2022**, *32*, 2202697.

[29] Q. Zhou, G. Yuan, K. Guo, S. Li, M. Lin, J. Hong, Y. Huang, *FlatChem* **2021**, *30*, 100303.

[30] T. Wu, Y. Xu, H. Wang, Z. Sun, L. Zou, *Carbon* **2021**, *171*, 639.

[31] H. Qiu, X. Zhao, H. Li, Y. Li, J. Li, J. Yang, *J. Appl. Polym. Sci.* **2021**, *138*, 51383.

[32] H. Huang, X. Ming, Y. Wang, F. Guo, Y. Liu, Z. Xu, L. Peng, C. Gao, *Carbon* **2021**, *180*, 197.

[33] H. Yuan, J. Ye, C. Ye, S. Yin, J. Li, K. Su, G. Fang, Y. Wu, Y. Zheng, M. Ge, R. Tang, G. Feng, Y. Qu, Y. Zhu, *Chem. Mater.* **2021**, *33*, 1731.

[34] Z. Pan, Y. Wu, H. Yuan, R. Tang, L. Ji, B. Zhou, C. Ye, D. Zhang, Y. Qu, H. Ji, Y. Zhu, *Carbon* **2021**, *182*, 799.

[35] G. Yang, H. Yi, Y. Yao, C. Li, Z. Li, *ACS Appl. Nano Mater.* **2020**, *3*, 2149.

[36] J. Li, J. Lai, J. Liu, R. Lei, Y. Chen, *Nanomaterials* **2020**, *10*, 531.

[37] R. Zou, F. Liu, N. Hu, H. Ning, X. Jiang, C. Xu, S. Fu, Y. Li, C. Yan, *Nanotechnology* **2020**, *31*, 065602.

[38] Y. Zhu, Q. Peng, Y. Qin, X. Zhao, L. Xu, Q. Chen, Y. Li, Z. Xu, X. He, *ACS Appl. Nano Mater.* **2020**, *3*, 9076.

[39] A. Akbari, B. V. Cunning, S. R. Joshi, C. Wang, D. C. CamachoMojica, S. Chatterjee, V. Modepalli, C. Cahoon, C. W. Bielawski, P. Bakharev, G. H. Kim, R. S. Ruoff, *Matter* **2020**, *2*, 1198.
[40] S. Chen, Q. Wang, M. Zhang, R. Huang, Y. Huang, J. Tang, J. Liu, *Carbon* **2020**, *167*, 270.
[41] X. Zhang, Y. Guo, Y. Liu, Z. Li, W. Fang, L. Peng, J. Zhou, Z. Xu, C. Gao, *Carbon* **2020**, *167*, 249.
[42] Y. Li, Y. Zhu, G. Jiang, Z. P. Cano, J. Yang, J. Wang, J. Liu, X. Chen, Z. Chen, *Small* **2020**, *16*, 1903315.
[43] J. Li, X.-Y. Chen, R.-B. Lei, J.-F. Lai, T.-M. Ma, Y. Li, *J. Mater. Sci.* **2019**, *54*, 7553.
[44] R. Zou, F. Liu, N. Hu, H. Ning, X. Jiang, C. Xu, S. Fu, Y. Li, X. Zhou, C. Yan, *Carbon* **2019**, *149*, 173.
[45] K. Wang, M. Li, J. Zhang, H. Lu, *Carbon* **2019**, *144*, 249.
[46] F. Xu, R. Chen, Z. Lin, X. Sun, S. Wang, W. Yin, Q. Peng, Y. Li, X. He, *J. Mater. Chem. C.* **2018**, *6*, 12321.
[47] X. Meng, H. Pan, C. Zhu, Z. Chen, T. Lu, D. Xu, Y. Li, S. Zhu, *ACS Appl. Mater. Interfaces* **2018**, *10*, 22611.
[48] H. Li, J. Miao, X. Wu, K. Cheng, H. Qiu, J. Yang, *J. Polym. Sci. B Polym. Phys.* **2018**, *56*, 1215.
[49] X. Chen, X. Deng, N. Y. Kim, Y. Wang, Y. Huang, L. Peng, M. Huang, X. Zhang, X. Chen, D. Luo, B. Wang, X. Wu, Y. Ma, Z. Lee, R. S. Ruoff, *Carbon* **2018**, *132*, 294.
[50] C. Teng, D. Xie, J. Wang, Z. Yang, G. Ren, Y. Zhu, *Adv. Funct. Mater.* **2017**, *27*, 1700240.
[51] J. Ding, H. Zhao, Q. Wang, H. Dou, H. Chen, H. Yu, *Nanoscale* **2017**, *9*, 16871.
[52] J. Ding, O. ur Rahman, H. Zhao, W. Peng, H. Dou, H. Chen, H. Yu, *Nanotechnology* **2017**, *28*, 39LT01.
[53] P. Kumar, F. Shahzad, S. Yu, S. M. Hong, Y.-H. Kim, C. M. Koo, *Carbon* **2015**, *94*, 494.
[54] Y. Zhang, H. Han, N. Wang, P. Zhang, Y. Fu, M. Murugesan, M. Edwards, K. Jeppson, S. Volz, J. Liu, *Adv. Funct. Mater.* **2015**, *25*, 4430.
[55] G. Xin, H. Sun, T. Hu, H. R. Fard, X. Sun, N. Koratkar, T. Borca Tasciuc, J. Lian, *Adv. Mater.* **2014**, *26*, 4521.
[56] B. Shen, W. Zhai, W. Zheng, *Adv. Funct. Mater.* **2014**, *24*, 4542.
